# Stimulus Intervals Modulate the Balance of Brain Activity in the Human Primary Somatosensory Cortex: An ERP Study

**DOI:** 10.3389/fninf.2020.571369

**Published:** 2021-01-27

**Authors:** Yang Liu, Bo Dong, Jiajia Yang, Yoshimichi Ejima, Jinglong Wu, Qiong Wu, Ming Zhang

**Affiliations:** ^1^Department of Psychology, Suzhou University of Science and Technology, Suzhou, China; ^2^Cognitive Neuroscience Laboratory, Graduate School of Natural Science and Technology, Okayama University, Okayama, Japan; ^3^Beijing Institute of Technology, Beijing, China; ^4^Department of Psychology, Soochow University, Suzhou, China

**Keywords:** traditional spatial attention paradigm, ERP, interstimulus interval, enhancement and suppression, primary somatosensory cortex

## Abstract

Neuronal excitation and inhibition occur in the brain at the same time, and brain activation reflects changes in the sum of excitation and inhibition. This principle has been well-established in lower-level sensory systems, including vision and touch, based on animal studies. However, it is unclear how the somatosensory system processes the balance between excitation and inhibition. In the present ERP study, we modified the traditional spatial attention paradigm by adding double stimuli presentations at short intervals (i.e., 10, 30, and 100 ms). Seventeen subjects participated in the experiment. Five types of stimulation were used in the experiment: a single stimulus (one raised pin for 40 ms), standard stimulus (eight pins for 40 ms), and double stimuli presented at intervals of 10, 30, and 100 ms. The subjects were asked to attend to a particular finger and detect whether the standard stimulus was presented to that finger. The results showed a clear attention-related ERP component in the single stimulus condition, but the suppression components associated with the three interval conditions seemed to be dominant in somatosensory areas. In particular, we found the strongest suppression effect in the ISI-30 condition (interval of 30 ms) and that the suppression and enhancement effects seemed to be counterbalanced in both the ISI-10 and ISI-100 conditions (intervals of 10 and 100 ms, respectively). This type of processing may allow humans to easily discriminate between multiple stimuli on the same body part.

## Introduction

When spatial attention to auditory (Alho et al., 1999; Karns and Knight, 2009) or visual stimuli (Noesselt et al., 2002; Macaluso et al., 2005) was modulated, evoked potentials were generated in the primary auditory or visual cortices. Regarding the somatosensory system, studies have been conducted using fMRI and event-related potentials (ERPs) in humans (Meador et al., 2002; Forster and Eimer, 2004; Schubert et al., 2008), and they found that attention enhances activity in the primary somatosensory cortex (SI) when using a single stimulus. Animal studies (Pilz et al., 2004; Braun et al., 2005; Reed et al., 2010) used double stimuli to show that the second stimulus suppresses the response to the first stimulus. This suggested that spatiotemporal interactions modulate the response magnitude in human SI. However, it remains unclear how the balance between attentional enhancement and double asynchronous stimulation-induced suppression is maintained.

Many previous studies examining the effects of spatial-selective attention have found that attentional effects occur in the early stage, but they did not find modulation of somatosensory evoked potential (SEP) components generated in S1. Some ERP studies used a mechanical tactile stimulus and found a contralateral N80 component with sustained attention and a bilateral P100 component with spatial attention in the early stages (Eimer and Driver, 2000; Eimer and Forster, 2003b; Zopf et al., 2004). Other electroencephalography (EEG) studies using tactile spatial sustained attention to mechanical stimuli found that the earliest somatosensory component (P50) was significantly increased for attended stimuli (Zopf et al., 2004). In a simultaneous EEG-fMRI study, Schubert et al. (2008) used Braille stimulation and found significant effects of spatial-selective attention on P50 and P100 with left tactile stimuli and on N80 with right tactile stimuli in SI. Other ERP and SEP studies of mechanical tactile stimuli (Eimer and Forster, 2003a; Eimer et al., 2004; Forster and Gillmeister, 2011; Katus et al., 2012) showed that amplitudes of mid-latency components such as N140 and P200 were enhanced in response to tactile stimuli presented to the attended hand.

In addition, an electrophysiological study in owl monkeys (Reed et al., 2010) selected paired skin sites and delivered pulses simultaneously (0 ms delay) or with onset asynchronies of 10, 30, 50, 100, and 500 ms to investigate the effects of varying the temporal proximity of stimuli. This study indicated that maximal suppression of firing rates occurred when the stimulus onset times were 30–50 ms. The owl monkeys were sedated in this study, so a suppressed effect was observed under unattended conditions.

The underlying attention and temporal processes in the human somatosensory cortex remain unclear when paired mechanical stimuli are presented. Thus, we hypothesized that enhancement and suppression occur as follows in human somatosensory areas: (1) The enhancement effect of sustained spatial attention will be stronger than the suppression effect of paired stimulation. (2) The suppression effect of paired stimulation will be stronger than the enhancement effect of sustained spatial attention. (3) The enhancement effect of sustained spatial attention and the suppression effect of paired stimulation will exist at the same time.

The present experiment was designed to determine whether the enhancement from sustained spatial attention or suppression from paired stimulation affects neurophysiological responses in human SI. We extended the work of previous studies to investigate the temporal dynamics of neural responses when mechanical tactile stimulation is delivered to the left or right index finger at different interstimulus intervals with attention focused on one hand. Participants were asked to focus their spatial attention on tactile stimulation of one hand (on a finger), and we instructed them to detect rare tactile target stimuli on the index finger of the attended hand. To achieve this aim, ERPs were computed in response to tactile stimulation.

## Materials and Methods

### Participants

Nineteen undergraduate students were recruited as volunteers. With further analysis, two participants were excluded from the statistical analysis because of low performance. Seventeen participants (age range: 21–25; mean age: 22.5) remained in the sample. All participants had normal or corrected-to-normal vision and were right-handed. They had no neurological/psychiatric disorders and no hearing problems. The experimental protocol was approved by the ethics committee of Okayama University.

### Material and Procedure

The experiment was conducted in a dimly lit, sound-attenuated room, with participants facing a computer screen (17 inch, LG, FLATRON) at a viewing distance of 60 cm. Tactile stimuli were applied to the distal phalanx of the left or right index finger using a piezoelectric Braille stimulator (KGS, Saitama, Japan). Each stimulator had eight individually controllable plastic pins grouped in a 2 × 4 array. The diameter of each pin was 1.3 mm. The distance between pins was 2.4 mm. Using a custom-built electrical drive, pins could be elevated from the resting position by 0.7 mm with a tactile force of 0.177 N/pin. The mechanical onset from the trigger to the highest position was ~38 ms, as measured by a high-speed camera, so we set the tactile stimuli presentation time to 40 ms.

**Figure 1:**
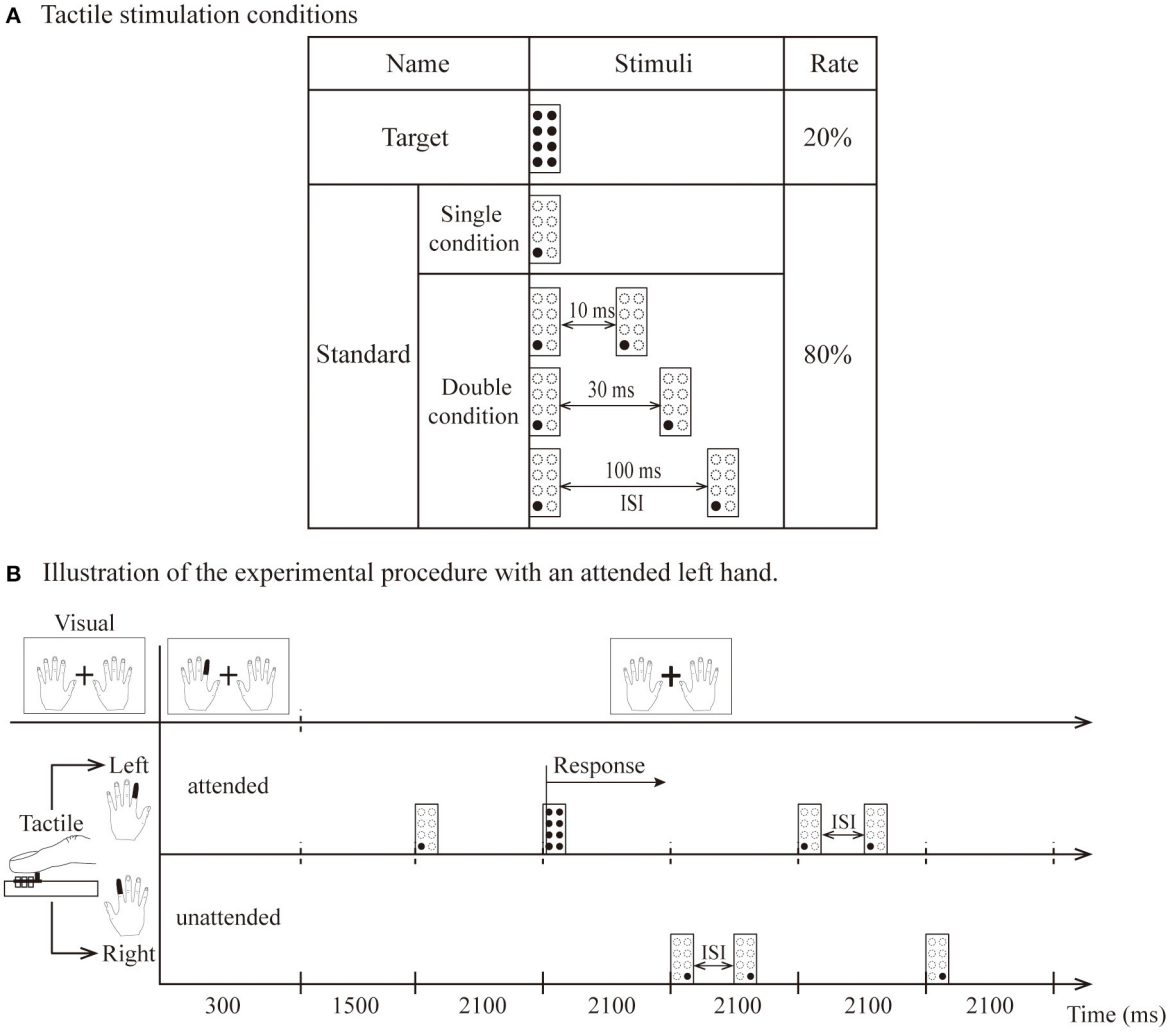
**(A)** The types of tactile stimulation: standard (1 pin) and target (8 pins). **(B)** Illustration of attended left hand. The visual instruction was presented for 300 ms, and participants were instructed to direct their attention to the left index finger until the next instruction appeared. Standard stimuli presented on the left hand as attended stimuli. Stimuli delivered to the other hand were unattended stimuli. After the 1,500 ms interval, tactile stimuli (including two target stimuli and eight standard stimuli per block) were presented unilaterally to the left or right hand within 600 ms (total 2,100 ms). The target was presented only on the left side, and the participant responded vocally when it was detected.

Tactile stimuli were included for the standard and target. The target was 8 pins and was presented only on the side indicated by the visual instructions. The standard was one pin in the lower left (or right) when stimuli were presented on the left (or right) index finger. The stimulus presentations were composed of single and double conditions. The temporal proximity of stimulus presentations in the double condition consisted of three different interstimulus intervals (10, 30, and 100 ms). The interstimulus interval (ISI) is the time interval between the first tactile stimulus offset and second tactile stimulus onset (Figure 1A).

Visual and tactile stimuli were presented by using Presentation software (Neurobehavioral Systems Inc., Albany, California, USA) housed outside of the dimly lit room. A block design was used for this experiment in which the standard and target stimuli were randomized in blocks of 10 trials, with 15 blocks in one session (for a total of 150 trials). In summary, the experiment comprised 16 separate sessions, consisting of 240 blocks for a total of 2,400 trials. Visual instructions indicating the left or right index fingers (each instruction angle was 5 × 7° flat at 3.5° left or right from the fixation) changed to red and were presented for 300 ms at the beginning of each block. The instructions asked the participants to keep their attention on the left or right index finger for that block. A fixation (a white cross of 1.7 × 1.7° of visual angle) was located between both instructions (Figure 1B). Each session contained four experimental conditions: a single condition and three types of double conditions (ISI-10 condition, ISI-30 condition and ISI-100 condition).

Figure 1B illustrates the experimental stimulation procedure for the attended left hand. Each block began with the visual instruction, which was presented for 300 ms. Within the 300 ms, the index finger of the left hand turned red in the visual instructions, and the subjects kept their attention on the finger position indicated by red (i.e., left index finger) until the next block. They were required to respond vocally when the target stimulus was detected on the left index finger. Thus, the participants had to direct their attention to the attended hand. A standard stimulus presented to this hand was named the attended stimulus. In contrast, stimuli delivered to the other hand were named unattended stimuli. After a 1,500-ms interval, tactile stimuli (including two target stimuli and eight standard stimuli per block) were presented unilaterally to the left or right hand within 600 ms (for a total of 2,100 ms as indicated in Figure 1B). Visual instructions and tactile stimulation were presented in pseudorandom order. During the entire experiment, the participants were also instructed to avoid movements of the body, in particular, the eyes and fingers.

### EEG Recording and Data Analysis

An EEG system (Brain Amp MR plus, Germany) was used to record signals through 28 electrodes mounted on an electrode cap (Easy cap, Herrsching Breitbrunn, Germany) as specified by the International 10–20 System. All electrodes were referenced to the combined signals from the bilateral earlobes. A horizontal electrooculogram (HEOG) was recorded from the outer canthus of the left eye. Eye blinks and vertical eye movements were recorded from an electrode placed 1.5 cm below the left eye. The impedance of all electrodes was below 5 kΩ. The raw signals were digitized with a sample frequency of 500 Hz with a 60-Hz notch filter. The bandpass of the amplifiers was DC to 250 Hz.

Brain Vision Analyzer software (version 1.05, Germany) was used to analyze the ERPs, which were averaged separately for each stimulus type offline. To remove the target stimulus, we analyzed only ERPs elicited by standard stimuli. The continuous EEG signals were segmented offline from 100 ms before to 500 ms after tactile stimulus onset. Baseline corrections were made against the data from −100 to 0 ms. We rejected artifact trials in which the amplitude reached ±80 μν from −100 to 500 ms, and we filtered the data with a bandpass filter retaining frequencies between 0.01 and 30 Hz. The data from each electrode were then averaged, and a grand average ERP was computed across all participants for each stimulus type.

For further analysis, the mean amplitude data were computed within the following time windows relative to stimulus onset: P50 (34–62 ms), N80 (64–92 ms), P100 (94–122 ms), N140 (124–172 ms), P200 (174–242 ms), and P300 (244–342 ms). In each time window, the mean amplitude data were analyzed using repeated measures analyses of variance (ANOVAs) with two factors (attended vs. unattended) × 4 conditions (single, ISI-10, ISI-30 and ISI-100 conditions), and data from electrodes C3 and C4 were analyzed separately. RStudio (Version 1.1.383) was used for all statistical analyses.

## Results

**Figure 2:**
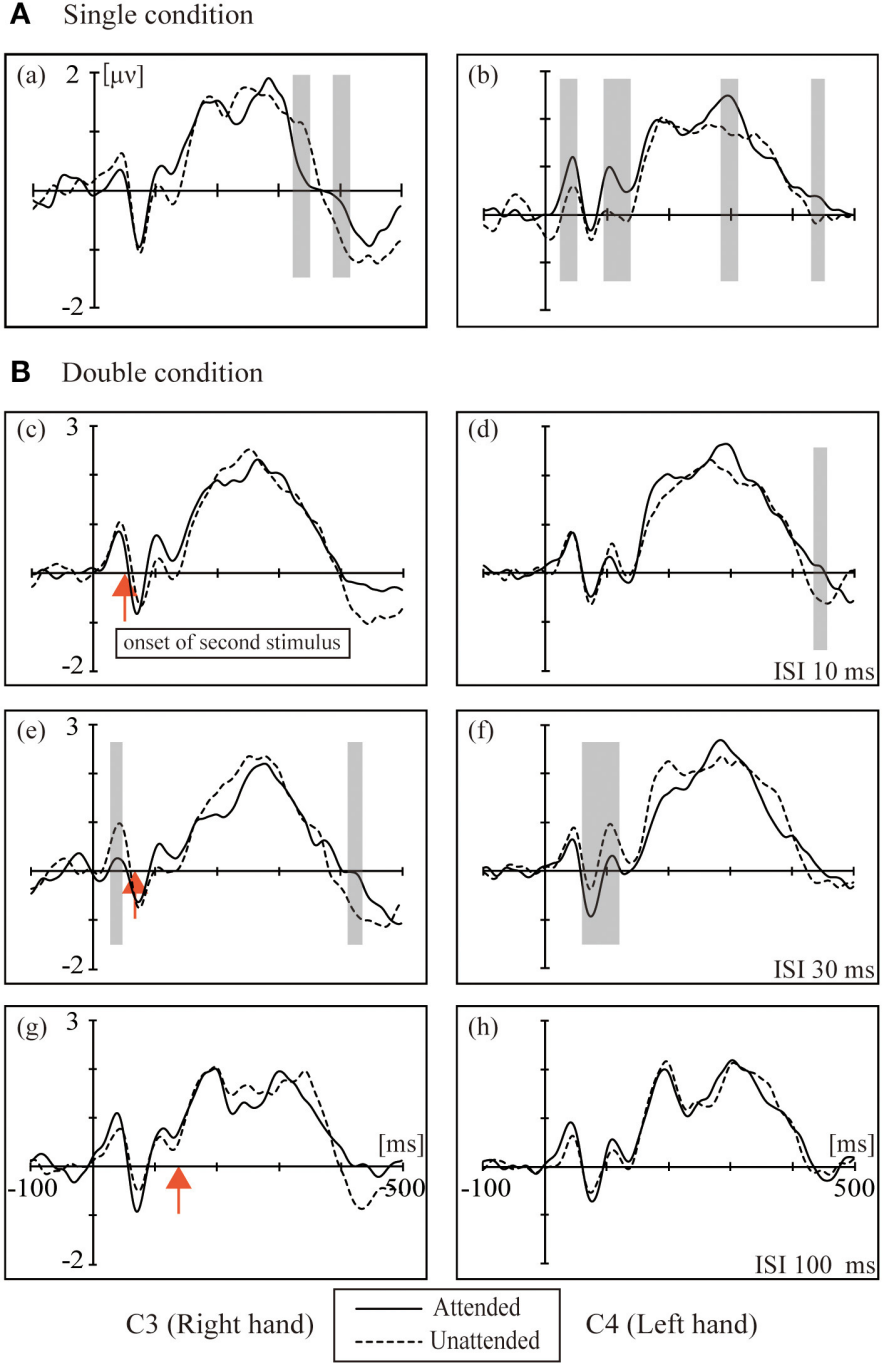
The grand averaged waveforms for the **(A)** single condition (a, b) and **(B)** double conditions: (c,d) ISI 10 ms; (e,f) ISI 30 ms; (g–f) ISI 100 ms. The electrode sites were C3/4 approximately overlying the contralateral SI. Black solid line: attended. Black dotted line: unattended. The red arrow marks the onset of the second stimulus. The shaded areas indicate the periods used for the pointwise running *t*-tests comparing attended to unattended for all participants (*p* < 0.05).

Figure 2 shows the grand averaged waveforms for the single condition and double conditions (ISI-10 condition, ISI-30 condition and ISI-100 condition). The electrode sites were C3/4, approximately overlying the contralateral SI. The black solid line represents the attended state, and the black dotted line represents the unattended state. In the single condition, attended stimuli elicited more positive responses than the unattended state. The double conditions resulted in the following: in the ISI-10 condition, the attended stimuli elicited activity similar to the unattended state; in the ISI-30 condition, the unattended stimuli elicited more positive activity than the attended state; and in the ISI-100 condition, the attended stimuli elicited activity levels close to the unattended state once again.

The left column in Figure 2 shows the ERPs elicited in the four conditions by tactile stimuli presented on the right index finger at contralateral electrodes (C3, right hand). All subjects demonstrated a clear P45 component in their responses to tactile stimuli presented to the right index fingers. ANOVA of the mean amplitudes of P45 revealed a main effect [*F*_(1,16)_ = 4.740; *p* < 0.05] of attention at C3, which was not accompanied by an attention × condition interaction; ANOVA of mean amplitudes of P100 revealed a main effect [*F*_(1,16)_ = 6.175; *p* < 0.05] of attention at C3, which was not accompanied by an attention × condition interaction. There was a main effect of conditions [*F*_(3,16)_ = 3.230; *p* < 0.05] at C3 for the P300 component.

The right column of Figure 2 shows the ERPs elicited in the four conditions by a tactile stimulus presented to the left index finger at contralateral electrodes (C4, left hand). The analysis of the left side for P45 and N80 revealed no main effect or interaction between attention and conditions, and only a weak significant difference in the *t*-test was found between the attention states in the ISI-30 condition (*p* < 0.05). There was a significant interaction between attention and conditions [*F*_(3,16)_ = 6.589; *p* < 0.001] for P100; paired *t*-tests found the most significant difference between the unattended and ISI-30 conditions (*p* < 0.001). No main effects of attention and conditions were found for N140, P200 and P300.

**Figure 3:**
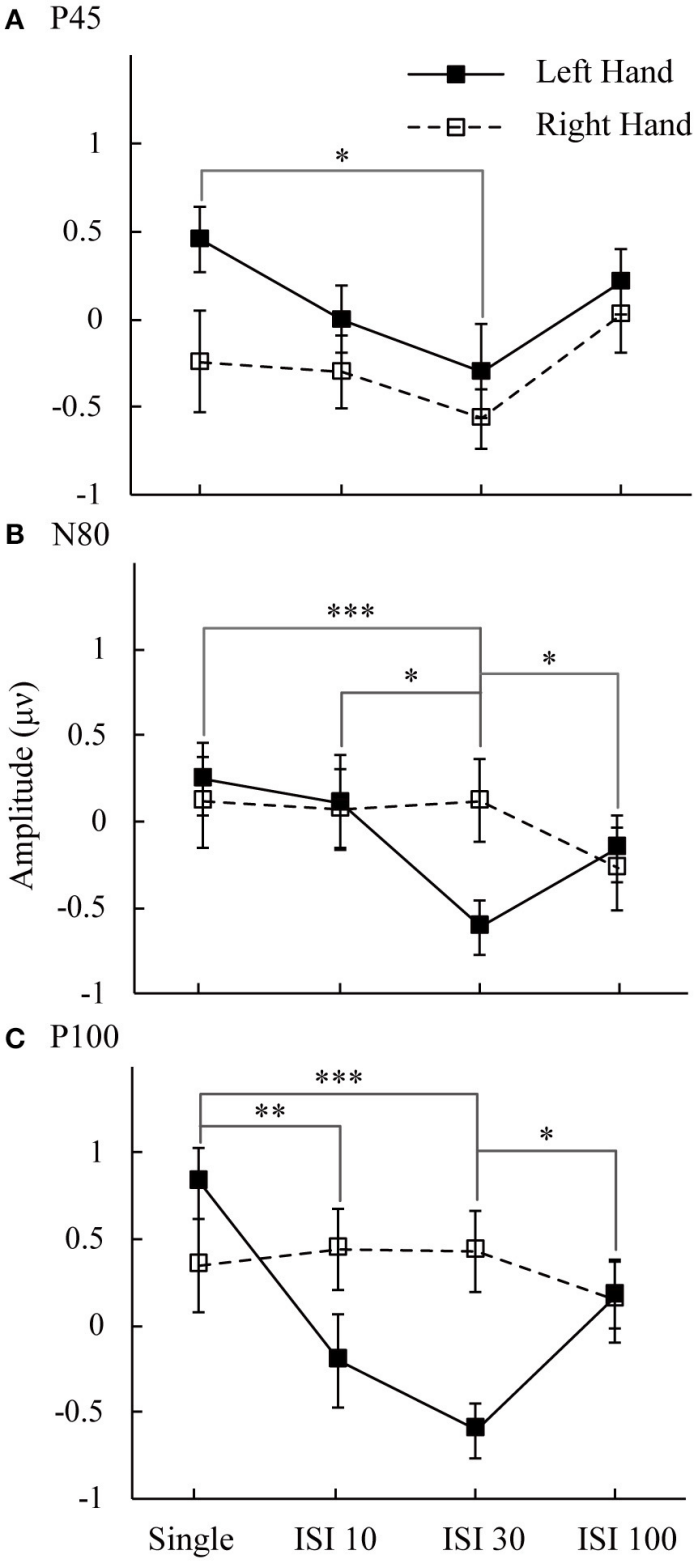
Mean amplitudes of attended minus unattended conditions on the left hand and right hand. The analysis time window for **(A)** P50 was 34–62 ms; **(B)** N80 was 64–92 ms; and **(C)** P100 was 94–122 ms. Black line: left hand. Dotted line: right hand. *p < 0.05, **p < 0.01, ***p <0.001.

Figure 3 shows the mean amplitudes for the P45, N80, and P100 components. This result represents the attended minus unattended conditions on the left hand and right hand. Three components showed the lowest amplitude in the ISI-30 condition with the left-hand stimulus. The main effect of attention on the mean amplitudes of the P45 component was significant [*F*_(1,16)_ = 6.14, *p* < 0.05]. *Post hoc* comparisons between the single and ISI-30 conditions showed that most activation occurred at the C4 electrode (*p* < 0.05). Regarding the N80 component, the interaction between attention and ISI was clear [*F*_(3,48)_ = 5.88, *p* < 0.05], and the mean amplitudes in the single and ISI-10 conditions were significantly higher than that in the ISI-30 condition (*p* < 0.05). ISI-30 and ISI-100 were also significantly different (*p* < 0.05). These results were also limited to the C4 electrode (left hand). In the last component, P100, there was no main effect or interaction at the C3 electrode, although an effect similar to the attention main effect was found [*F*_(1,16)_ = 3.77, *p* = 0.07], but at the C4 electrode, an interaction effect between attention and ISI was clearly found [*F*_(3,48)_ = 6.6, *p* < 0.05]. The mean amplitude in the single condition was higher than that in the ISI-10 and ISI-30 conditions (*p* < 0.01). Additionally, there was a significant difference between ISI-30 and ISI-100 conditions (*p* < 0.05). For the right hand, there were no significant differences between conditions for P45, N80, and P100.

## Discussion

This study used double asynchronous stimulation to investigate the relationship between spatial attention enhancement and double asynchronous stimulation-induced suppression of brain activity in human SI. The participants were asked to focus their spatial attention on tactile stimulation of one hand (on a finger), and we instructed them to detect rare tactile target stimuli at the index finger of the attended hand. In double stimulation conditions, as the interstimulus intervals increased, a V-shaped effect was observed. We suggest that this occurs through an attention enhancement and a double stimulation-suppression effect.

We found a suppression effect in the ISI-30 condition, supporting a hypothesis from previous studies that multisensory stimulation shortens the response latencies of neurons and that post-activation inhibition of neurons is stronger than single stimulation. Research in monkeys found that the suppressive effect of paired stimulation activation on the interphalangeal nerve was stronger than that on the adjacent phalanges. The inhibition of interphalangeal nerve activity was caused by the proximity of receptor-related nerve cells in area 3b, which leads to nerve post-activation inhibition. As was observed in monkeys, neural response intensity was generally suppressed by a preceding conditioning stimulus when the test stimulus occurred after a 30- or 50-ms delay (Reed et al., 2010). Other similar studies (Fanselow and Nicolelis, 1999) examining rat whisker nerve reflexes found nerve post-activation inhibition following paired stimulation in quiet and movement states. In addition, Christian 2017 used double visual stimuli to investigate repetition suppression and suggested that stimulus-specific expectations about objects modulated the LOC and propagated back to the earliest cortical station processing visual input (Grill-Spector and Malach, 2001; Utzerath et al., 2017). In the present study, the visual input was equivalent to cues to improve sensitivity to the tactile input, and the stimulus was repeatedly presented in the same location of the fingers. It was more intuitive to find nerve post-activation inhibition in area 3b. This experiment extended previous studies in monkeys and verified that the paired stimulation suppression effect in human primary somatosensory cortex 3b is similar to that in monkeys. The time of nerve post-activation inhibition may be ~30–50 ms.

In the single condition, we found some significant ERP components in the contralateral hemisphere by comparisons with the unattended side. The P50 and P100 components at the C4 electrode were significantly stronger on the attended side than on the unattended side (Figure 2). An fMRI-EEG study used braille stimulation to investigate attentional effects on S1, and it found that left tactile stimulation (P50) was significantly enhanced by spatial-selective attention, suggesting that attention enhances the sensory signal during its early passage in S1(Schubert et al., 2008). This study also showed that P50 was the earliest component to be modulated by spatial-selective attention using stimuli similar to braille stimulation. Thus, the asymmetric effects of spatial selective attention on the two sides could also be found in the early and middle processing stages. For stimuli on the left hand, P50, P100, and P300 were found when comparing the attended vs. unattended hand, but on the other side, only the P300 attentional effect was found in the attended vs. unattended hand comparison. These asymmetric hemispheric activations may be explained by Mesulam's modality-non-specific model of spatial attention (Mesulam, 1999). That is, higher-order areas in the left hemisphere control attention for events only on the right side, whereas the right hemisphere controls attention for both the left and right sides. Both theories may explain the asymmetric attentional effects on the SEPs, leading to earlier attentional modulation for left stimuli (i.e., P50 and P100 only for left and not for right stimuli).

We found some attentional enhancement in the single condition only. In the double stimuli conditions, the attentional effect was partially decreased as the interstimulus interval increased. A previous study suggested that when two or more stimuli were presented, the inhibition effects in based on the preferred stimulus (Reed et al., 2010). In the ISI-10 condition, we did not observe any enhancement or suppression effect. There are two possibilities that explain these results: the interval may be too short, such that the subject cannot recognize the double stimuli, and when the stimulus is changed to double, the suppression effect is activated much more strongly than the attentional enhancement effect. According to the interaction of spatial attention enhancement and double asynchronous stimulation-induced suppression, when the enhancement and suppression effects are equal, there was no difference between attended and unattended states in terms of the neurophysiological responses to double asynchronous stimulation (Figures 2, 3). We suggest a tentative explanation that may account for this finding: the attention enhancement and double asynchronous stimulation-induced suppression effects decreased as the interstimulus interval increased. The stimulatory effect of attention is mutually competitive with the inhibitory effect of double stimulation. Moreover, the enhancement of spatial attention may be modulated by double stimulation suppression.

## Data Availability Statement

The raw data supporting the conclusions of this article will be made available by the authors, without undue reservation.

## Ethics Statement

The studies involving human participants were reviewed and approved by the Ethics Committee of Okayama University. The patients/participants provided their written informed consent to participate in this study.

## Author Contributions

YL, BD, JY, JW, QW, and MZ designed experiments. YL, BD, JY, YE, JW, and QW conducted experiments. YL and BD analyzed data. YL, BD, QW, and MZ wrote manuscript. All authors approved the manuscript.

## Conflict of Interest

The authors declare that the research was conducted in the absence of any commercial or financial relationships that could be construed as a potential conflict of interest.
